# Cholera in Haiti and Other Caribbean Regions, 19th Century

**DOI:** 10.3201/eid1711.110958

**Published:** 2011-11

**Authors:** Deborah Jenson, Victoria Szabo, the Duke FHI Haiti Humanities Laboratory Student Research Team

**Affiliations:** Duke University, Durham, North Carolina, USA

**Keywords:** Haiti, cholera, Vibrio cholerae, bacteria, Cuba, Jamaica, Caribbean, epidemic, nineteenth century, journalism, slavery, military, colonialism

## Abstract

Medical journals and other sources do not show evidence that cholera occurred in Haiti before 2010, despite the devastating effect of this disease in the Caribbean region in the 19th century. Cholera occurred in Cuba in 1833–1834; in Jamaica, Cuba, Puerto Rico, St. Thomas, St. Lucia, St. Kitts, Nevis, Trinidad, the Bahamas, St. Vincent, Granada, Anguilla, St. John, Tortola, the Turks and Caicos, the Grenadines (Carriacou and Petite Martinique), and possibly Antigua in 1850–1856; and in Guadeloupe, Cuba, St. Thomas, the Dominican Republic, Dominica, Martinique, and Marie Galante in 1865–1872. Conditions associated with slavery and colonial military control were absent in independent Haiti. Clustered populations, regular influx of new persons, and close quarters of barracks living contributed to spread of cholera in other Caribbean locations. We provide historical accounts of the presence and spread of cholera epidemics in Caribbean islands.

## Abstract 2

Epidemic cholera did not occur in Haiti before 2010.

Research indicates that “*Vibrio cholerae* was newly introduced into Haiti” in 2010 (*1*). Yet, as recently as July 2011, the Boston Globe claimed that “Cholera appeared in Haiti last year for the first time since the 1960s” (*2*), implying that cholera had occurred in Haiti in the 20th century. The uncertainty in newspapers and nongovernmental organizations over whether Haiti had experienced cholera during the 20th century, a period when there was no epidemic cholera in the Caribbean islands, reflects the historically uneven documentation of cholera epidemiology in different regions.

Almost 2 centuries of cholera research such as John Snow’s famous environmental detective work on cholera in mid 19th century England (*3*) helped to found the field of epidemiology. However, until 2011, there was no comprehensive historical record of cholera in Africa (*4*), despite its crucial status as a piece of the global disease puzzle. Epidemiologic frameworks must nevertheless confront the transnational influences and fluid biosocial networks that influence the global routes of epidemic pathologies (*5*). The cultural and linguistic heterogeneity of the Caribbean island states, many of which were ruled by consecutive or simultaneous European colonial dominions, amid dwindling Native American populations and huge forced influxes of Africans of diverse ethnicities, is paradigmatic of the challenge for epidemiology of searching beyond the narratives privileged by a given institutional structure, such as the colonial medical research apparatus.

## Historical Context for Cholera in Haiti

Although it is difficult to confirm the absence of a disease in an earlier era, this report explores textual evidence for the immunologic status of persons to cholera in Haiti reported in scientific journals and lay journalism sources from the 19th century. It also considers the reasons that epidemic cholera failed to occur in Haiti in the 19th century even though it did affect many neighboring environments, including the Dominican Republic.

There is no published source that identifies all Caribbean islands affected by cholera epidemics in the 19th century. In the field of 20th-century and 21st-century research, an article in 1985 by Kiple comes the closest to describing the effect of cholera across the 19th-century Caribbean. In assessing Cuba, Jamaica, Puerto Rico, and St. Thomas, Kiple proposed that 200,000 deaths from cholera in the Caribbean would not be unrealistic, although a total figure would have to be “considerably higher as a number of islands have not been included in this survey” (*6*). Higman notes that basic public health measures were prompted by cholera epidemics in the British Caribbean in the 19th century but that they emerged from “a maze of environmental mystery” (*7*). Vega Lugo provides a recent study of cholera in 19th-century Puerto Rico (*8*).

However, the 3 major overviews of cholera in the 20th century and 21st century, the book Cholera by Pollitzer in 1959 (*9*), the foundational work with the same title by Barua and Greenough in 1992 (*10*), and the Encyclopedia of Pestilence, Pandemics, and Plagues by Byrne in 2008 (*11*), do not compare the timeframe of the totality of Caribbean epidemics in the 19th century with those of global pandemics or their historically debated environmental influences such as hurricanes and drought. When cholera occurred in Peru in 1991 but did not spread from Latin America to Caribbean islands, researchers may have asked different questions than they would have otherwise (*12*), although their concluding assessment and prescriptions remained accurate: “The lack of reported cases in Uruguay and the Caribbean may reflect a low risk for ongoing transmission...” (*13*). From the time of the earliest medical commentaries on epidemics in the Caribbean, researchers had noted that proportions of infections and deaths were “much higher than in all European cholera epidemics” (*14*).

In the 19th century, there was abundant discussion of Caribbean cholera in international medical journals such as Lancet or Gazette Médicale de Paris, and in colonial medical and political reports to European colonial dominions. However, cultural and political heterogeneity in the Caribbean discouraged 19th-century researchers from providing a comparative overview of Caribbean cholera epidemics. Furthermore, their reigning hypotheses, such as the noncontagion theory, and their motivations, such as proslavery or antislavery arguments, differed from those of medical and research establishments today. Considerable variation in accounts of the numbers of the sick and the dead was the rule rather than the exception.

Fortunately, where no complete narrative exists, searchable databases (e.g., Digital Collections of the US National Library of Medicine or Google Books for medical sources, and America’s Historical Newspapers, World Newspaper Archives, or Gallica for journalism sources) now provide the capacity to research many fragments. In the 19th century Caribbean context, it is useful to compare and contrast medical and lay sources. Journalism in the 19th century attempted to ensure successful navigation of culturally diverse environments through scoops from traveling merchants and maritime personnel.

These unwitting citizen participants in epidemiologic journalism about the Caribbean had the simple but compelling goal of preserving maritime commerce from contamination, quarantines, and disease-devastated economies through direct testimony about epidemics. In a typical example, readers learned of cholera in Puerto Rico through the testimony of a Newburyport captain who was thwarted in his effort to do business in the harbor.

“Capt. Reed, of the schooner Life Boat, at Newburyport, reports that the cholera was raging fearfully at Mayaguez, carrying off from 40 to 50 persons daily. The epidemic broke out about the 5th of August, and when Capt. R. left was rather on the decrease. It had stopped business entirely, so that very few sales or purchases could be made, and laborers could with difficulty be obtained. Capt. R. carried out spars on deck, and says that the men employed to float them ashore were all dead within 4 hours of the job being completed” (*15*).

The Caribbean region experienced cholera in 3 major waves, mostly overlapping the timeframes of Barua and Greenough (*10*) for the second (1829–1851), third (1852–1859), and fourth (1864–1879) cholera pandemics. The 3 periods of cholera in the Caribbean that we have identified are 1833–1834 (with, according to Kiple [*6*], possible lingering cholera in outlying areas until late 1837 or early 1838) in Cuba; 1850–1856 in Jamaica, Cuba, Puerto Rico, St. Thomas, St. Lucia, St. Kitts, Nevis, Trinidad, the Bahamas, St. Vincent, Granada, Anguilla, St. John, Tortola, the Turks and Caicos, the Grenadines (Carriacou and Petite Martinique), and possibly Antigua; and 1865–1872 in Guadeloupe, Cuba, St. Thomas, the Dominican Republic, Dominica, Martinique, and Marie Galante.

## Cholera in the Caribbean Region

Cholera reached the Western Hemisphere during the second pandemic, although the term cholera morbus was in use much earlier to designate a range of conditions including dysentery and inflammation and bleeding in the digestive tract. Thus, an 1816 public health report in Baltimore read, “There died in Baltimore, in the year 1815, 1349 persons, among them 218 of consumptions; 167 of Cholera Morbus; 108 of pleurisy; 98 of fits; 83 of old age; 74 of worms; … 3 of cancer” (*16*). A 1783 obituary in Boston read, “[H]e made a collection of Toad-stools, under the notion of Mushrooms, which, having fryed they eat the following evening; but … their supper proved a poison to them, operating much like a Cholera Morbus, of which said Kreamer expired” (*17*). Although researchers have remained cautious about distinguishing between such earlier uses of cholera morbus and the cholera associated with pandemics, lay journalism sources in June 1832 in Quebec, Montreal, and New York had no qualms about distinguishing ordinary cholera, common at this season of the year, from the ravages of the Asiatic cholera (*17*). Asiatic cholera, associated with “large numbers of Irish and other emigrants,” a “class of persons particularly exposed and carrying the disease wherever they go,” killed considerable numbers of persons <6 hours from onset of symptoms. Rosenberg noted that in the context of cholera in the United States, “the newly arrived immigrant found all doors closed to him” (*18*).

Cholera arrived in the Caribbean in late February (24 or 25), 1833 (*4**,**19*). The first reported death was of a Catalan man named José Soler who lived in the Lazaro neighborhood of Havana, Cuba, in the poor port section outside the city walls. A neighboring mulatta woman then died, followed by the nearly mass extinction of barracks of newly arrived slaves from Africa, leading to the rumor that they had brought cholera with them. However, this rumor was likely not true because cholera did not arrive in West Africa until 1868 in Senegal and 1869 in the Gambia (*4*). Day by day, cholera spread through different neighborhoods of Havana. Cholera spread from Havana to the Matanzas region in mid-March and continued to rage there until mid-June. After an apparent pause in large-scale epidemic activity, cholera then flared up in Havana and Trinidad (Cuba), in the summer and fall of 1834.

The severe death toll in 1833–34 was a first harbinger of a disastrous relationship in the Caribbean between cholera epidemics and the 2 major imported populations typically housed in barracks, often weakened in advance of their arrival by shipboard conditions and after their arrival by the encounter with yellow fever and plague: slaves and colonial soldiers. We read that in Matanzas, “Two cargoes of slaves (over 1000), arrived a few days since; … all of whom died” (*20*). In August, another large group of newly arrived slaves heard such stories, “which drove the newcomers to desperation, and thinking that they might as well die in one way as another, they rose upon their keepers and murdered them” (*21*).

Even when cholera briefly waned Cuba in late June 1833, the community was wary of what the future held. They asked “Where is our guarantee that the disease is not located permanently in the island? How is confidence utterly lost and ruined to be restored?” (*22*).

After the first wave of Caribbean cholera, the epidemic reappeared in Cuba in February 1850 but not as severe as in 1833. According to a European medical bulletin, the Health Office in Havana had first confirmed the epidemic in a specific location (the military hospital). However, outside the military community, the effects seemed mild: the Daily National Intelligencer noted that among citizens, after the first 3 weeks of the epidemic, “panic has in great measure subsided” (*23*). In an intriguing but indirect description of what may have reflected effects of partial local immunity resulting from recent outbreaks on the severity of the disease, this article claimed that “for cholera to become fatal, the patient must have an elective affinity for the disease.”

In the first cholera epidemic in Jamaica in the fall of 1850, there was no necessary elective affinity for patients to court death. Some medical observers claimed that it had taken the lives of 10% of the total population by the time it subsided in Kingston in early January 1851 (*24*). Unpredictable movements from area to area kept the epidemic in Jamaica going for 2 years. Although there was some overlap of affected communities in 2 most recent of these regional epidemics, new influxes of soldiers may have accounted for regional epidemics, as they apparently did for flare-ups in Kingston: “The soldiers in camp at Kingston had suffered severely” (*25*). Cholera would occur again in Jamaica during April–September 1854, mostly in regions (Mont Diablo, Sturgetown, Salem, and St. Ann’s Bay) not devastated in previous epidemics.

During February–March 1851, Cuba experienced a pattern of sporadic and mild cases in Havana. However, during late September–December 1852, Santiago was affected with an appalling intensity. This regional extremity of the island had not been struck by cholera in the 1830s, and its population was therefore still immunologically naive: “The town of St. Jago de Cuba experienced its first and only visitation in 1852” (*26*).

Beginning in late September 1852, cholera devastated Nassau in the Bahamas. The Daily Atlas described “Vessels in the harbor being crowded with persons fleeing from the scourge” (*27*).

In 1853, cholera reappeared in Cuba in specific demographic groups. One journalist spoke of the toll among newly arrived slaves and soldiers: “The thousands of recently introduced Africans have brought with them a terrible kind of diarrhea, which is carrying off vast numbers of victims…. In Havana the troops are said to be dying like rotten sheep” (*28*).

These cholera episodes were only a prelude to 1854, when cholera epidemics exploded across the Caribbean. This was a period in which “immigrant vessels” from Europe were identified as catalysts. However, an east-to-west pattern for the movement of the epidemics, especially along the chain of the Windward Islands, may be indicative of more local transmission. Island after island saw its already small population reduced. The Albany Evening Journal recounted, “The arrival of the steamer Curlew, from St. Thomas, brings detailed accounts of the ravages of the cholera in those islands. Of the 14,000 inhabitants of the island of St. Thomas, 1,600 died. At Tortola, there were not enough survivors to bury the dead. At Nevis, out of 5,000, there have been 550 deaths” (*29*). In Barbados, which had a total population of 126,000, cholera exacted an appalling toll. Accounts from Bridgetown described the disposal of bodies as resembling heaps of merchandise consigned to the grave.

Although many communities appeared to recover from the epidemics, this was not always the case. In Trinidad in 1853, cholera has been blamed for the disappearance of Native Americans on the northern coast of this island (*30*). (Unfortunately, there is little information about this aspect of the epidemic because a curious phenomenon, that of hundreds of monkeys falling dead from the trees of what was believed to be cholera, dominated journalistic and medical commentary [*31*].)

In Puerto Rico, awareness of a surrounding Caribbean world destabilized by cholera led to a host of preventative measures ranging from quarantines to spraying the mail with vinegar (*6*). However, in November 1855, the disease belatedly arrived.

After the end of the Puerto Rico epidemic in 1856, there was a calm of almost a decade. However, in 1865, cholera was reimported by a ship from cholera-stricken Marseille to Guadeloupe, launching an epidemic that would exact a toll of >11,000 deaths in a population of 150,000. Martinique, Marie Galante, and Dominica were also affected, but sparsely.

In 1868, cholera returned to its Caribbean starting point, which was now a major battlefield of the Cuban revolution. For a time, cholera and yellow fever brought the hostilities to a halt, in a kind of pathologic truce. In Nuevitas in early August 1869, cholera reportedly caused the deaths of 200 Spanish troops a day. It had waned in Puerto Principe in late August, leading the Spanish leader to call for a general public thanksgiving. One journalist commented that because the rebels had left the area to avoid being infected, “Perhaps, in offering thanksgiving for the disappearance of cholera, the Spaniards may be celebrating the occupation of their district by the Rebels” (*32*). In 1870, there was cholera in Santiago and Havana; in 1871, cholera was reported in urban slums and among Spanish troops. A final possible epidemic was reported in 1872.

## The Exception in Haiti

In the 3 pandemics that involved the Caribbean in the 19th century, we found no medical or lay reports of cholera in Haiti, the Netherlands Antilles of Aruba, Bonaire, and Curaçao; the Cayman Islands; and St. Martin, St. Barthélemy, or Montserrat. (Martinique is a borderline case because despite reports of 1 or 2 cases of cholera in 1835, and sparse epidemic effect in 1865 and 1866, cholera never caused large-scale mortality rates there.) Of these sites, Haiti is the largest and most densely populated and was a close neighbor of hard-hit Cuba and Jamaica. Journalistic coverage in Haiti of cholera epidemics elsewhere in the official paper La Feuille du Commerce make it unlikely that epidemic cholera occurred there but went undocumented.

In the decades leading into the Caribbean era of cholera, the cultural situation in Haiti was unique. The Haitian revolution, beginning with the slave insurrection of 1791, led to the defeat of French colonialism and the founding of the first independent nation of former slaves in 1804 (*33*). (The course of the Haitian Revolution was influenced by epidemic yellow fever, a disease in relation to which Haiti was cited in a broad array in medical texts in the 19th century.) After 1804, Haiti continued to be a major trade partner with the United States and many other nations, even during embargo periods, as attested by lists of international ships in the harbor of Port-au-Prince on the front page of every issue of La Feuille du Commerce. However, key elements of integration of diverse world populations in substandard living conditions were absent in Haiti because of the lack of plantation slavery and colonial military troops.

Government records in Haiti from the 19th century show early recognition of the cholera threat posed by maritime traffic with Europe and the United States. In August 1832, even before the arrival of cholera in the Caribbean, Haitian President Boyer urged the adoption of a *cordon sanitaire* in port communities:

“Prudence dictates that we take all necessary means to prevent, to the extent that it is possible, the invasion of Haiti by cholera morbus, which has already traversed Europe and penetrated the United States. I urge you all… to take the most appropriate local measures with regards to ships arriving from ports in the United States, whose geographic proximity with Haiti makes the danger of epidemic spread particularly acute” (*34*).

Even in the fourth pandemic and in the most recent epidemics of cholera in the 19th century, when cholera did not spread into the Caribbean, the government in Haiti took measures to prevent introduction of cholera. In an 1852 English medical report (*35*), glancing allegation of cholera in Haiti was made in a long list of worldwide outbreaks. However, this reference appears to be inaccurate because an 1851 epidemic of malignant gastritis in Haiti was characterized by rotting of the stomach, a symptom that is not characteristic of cholera. A contemporary US newspaper article also discredited the rumor of cholera in Haiti from 1851: “From Aux Cayes [Haiti]. The schooner Panama from Aux Cayes reports that port as healthy. The rumor that cholera was raging proves false” (*36*).

Haiti did act when the neighboring Dominican Republic on the island of Hispaniola had a cholera epidemic, apparently imported from St. Thomas, beginning in late 1867. On March 14, 1868, the New York Herald reported “The Puerto Rican health boards have declared all Dominican ports foul on account of cholera. No vessels coming from thence will be allowed entry” (*37*), but Haiti had already established a policy of quarantining ships from the eastern side of the island. On April 1, 1868, a *Te Deum* was sung in Santo Domingo in thanks for the disappearance of the cholera that had been decimating the population, but there was never any sign of spread into Haiti (*38*).

Haiti did not participate in a system of centralized international public health reporting on infectious epidemics until 1881, when Haitian delegate Stephen Preston, Envoy and Minister Plenipotentiary at Washington, voted for the following resolution at the international Sanitary Conference.

“A centralized international system of sanitary notification being deemed indispensable to the successful carrying out of measures for preventing the introduction of disease, it is advisable to create international organizations to be charged with the duty of collecting information in regard to the outbreak, spread, and disappearance of cholera, pest, yellow fever, etc., and of conveying such information to the parties interested” (*39*).

However, Haiti historians had already been engaged in reporting the striking absence of cholera there, as in this late 1850s comment by the eminent Haitian historian Thomas Madiou:

“It must be observed that cholera has never entered in Haiti, even when it raged all around our island, in St. Thomas, Puerto Rico, Jamaica, and Cuba, in the Lesser Antilles and the Greater Antilles alike. Could this be due to some emanations of our soil that don’t allow choleric toxins to survive, or to some condition of our atmosphere?” (*40*).

The absence of earlier epidemic cholera in Haiti contrasts with the presence of epidemic cholera in the Caribbean in the 19th century. A digital map (www.caribbeancholera.org) shows how major cholera outbreaks unfolded over time in the Caribbean region. The 3 key clusters during 1833–1834, 1850–1856, and 1865–1872 are documented with newspaper and other historical accounts of the spread of cholera among and within countries in the Caribbean in the 19th century. Haiti did not have any cholera outbreaks in the 19th century, even when this disease was raging in surrounding regions. At that time, Haiti did not have the risk factors of imported new populations of slaves and colonial soldiers, which had been devastating in Cuba. Cuba, with its repeated outbreaks over the 19th century, was the last country in the Caribbean to abolish slavery (in 1879) and one of the last to overthrow European colonial dominion. In the contemporary epidemic in Haiti, influxes of international soldiers from cholera-endemic areas, who lived in barracks-style housing, present an unexpected parallel to the 19th-century risk factor of new colonial enslaved or military populations living in crowded conditions.
